# Ubiquitous Expression of *MAKORIN-2* in Normal and Malignant Hematopoietic Cells and Its Growth Promoting Activity

**DOI:** 10.1371/journal.pone.0092706

**Published:** 2014-03-27

**Authors:** King Yiu Lee, Kathy Yuen Yee Chan, Kam Sze Tsang, Yang Chao Chen, Hsiang-fu Kung, Pak Cheung Ng, Chi Kong Li, Kam Tong Leung, Karen Li

**Affiliations:** 1 Department of Paediatrics, The Chinese University of Hong Kong, Hong Kong; 2 Department of Anatomical and Cellular Pathology, The Chinese University of Hong Kong, Hong Kong; 3 Centre for Emerging Infectious Diseases, Department of Medicine and Therapeutics, The Chinese University of Hong Kong, Hong Kong; Emory University, United States of America

## Abstract

Makorin-2 (MKRN2) is a highly conserved protein and yet its functions are largely unknown. We investigated the expression levels of *MKRN2* and *RAF1* in normal and malignant hematopoietic cells, and leukemia cell lines. We also attempted to delineate the role of *MKRN2* in umbilical cord blood CD34^+^ stem/progenitor cells and K562 cell line by over-expression and inhibition of *MKRN2* through lentivirus transduction and shRNA nucleofection, respectively. Our results provided the first evidence on the ubiquitous expression of *MKRN2* in normal hematopoietic cells, embryonic stem cell lines, primary leukemia and leukemic cell lines of myeloid, lymphoid, erythroid and megakaryocytic lineages. The expression levels of *MKRN2* were generally higher in primary leukemia samples compared with those in age-matched normal BM cells. In all leukemia subtypes, there was no significant correlation between expression levels of *MKRN2* and *RAF1*. sh-MKRN2-silenced CD34^+^ cells had a significantly lower proliferation capacity and decreased levels of the early stem/progenitor subpopulation (CFU-GEMM) compared with control cultures. Over-expression of *MKRN2* in K562 cells increased cell proliferation. Our results indicated possible roles of *MKRN2* in normal and malignant hematopoiesis.

## Introduction

Makorin-2 (HSPC070; MKRN2) belongs to the *MKRN* gene family of which the ribonucleoproteins are characterized by a variety of zinc-finger motifs [1], [2]. MKRN2 holds four C_3_H zinc fingers and a signature C_3_HC_4_ RING zinc finger domain. *MKRN2* is a highly conserved gene [1] yet its function remains largely unknown. Previous studies reported that mkrn2 in *Xenopus laevis* acted upstream of glucogen synthase kinase-3β in the phosphatidylinositol 3-kinase/Akt pathway. The third C_3_H zinc finger and the RING motif are required for the anti-neurogenesis activity [3], [4]. *MKRN2* was first identified in human CD34^+^ stem/progenitor cells, as well as in some leukemic cell lines [2], [5], [6]. In chromosome 3p25, *MKRN2* is located next to the proto-oncogene *RAF1*. Interestingly, they share a sequence of 105 bp in the 3' UTR in a reversed transcription orientation [2]. This antisense sequence-overlapping of *MKRN2* with *RAF1* suggested that these two genes may regulate each other and be involved in normal hematopoietic and leukemic development. In this study, we investigated the expression levels of *MKRN2* and *RAF1* in normal and malignant hematopoietic cells, and leukemic cell lines. We also attempted to explore the role of *MKRN2* in umbilical cord blood CD34^+^ stem/progenitor cells and K562 cell line by over-expression and inhibition of *MKRN2* through lentivirus transduction and shRNA nucleofection, respectively. Our results demonstrated ubiquitous mRNA expression of *MKRN2* and *RAF1* in normal hematopoietic cells, embryonic stem cell lines, primary leukemia and leukemic cell lines. We also showed *MKRN2* functions on promoting cell proliferation of primary CD34^+^ progenitor cells and K562 cells, indicating its possible involvement in normal and malignant hematopoiesis.

## Materials and Methods

### Ethics statement

Written informed consents were obtained for collection of all human samples. For minors/children enrolled in the study, written consents were obtained from their parents on their behalf. This study was approved by the Ethics Committee for Clinical Research of The Chinese University of Hong Kong. All necessary permits were obtained for the described study, which complied with all relevant regulations.

### Patients and samples

Primary leukemic cells (over 70% blast cells) were obtained from the bone marrow of children (age ≤19 years) who were newly diagnosed with chronic myeloid leukemia (CML), acute lymphoid (ALL) or acute myeloid (AML) leukemia at the Prince of Wales Hospital, The Chinese University of Hong Kong. Age-matched normal subjects were siblings of patients who donated bone marrow for transplantation.

Peripheral blood samples were collected from normal adult volunteers. Mononuclear cells (MNC) were enriched by Ficoll-Hypaque density gradients (Amersham, Piscataway, NJ, USA). Human umbilical cord blood (CB) MNC and enriched CD34^+^ cells were obtained from full-term deliveries as described previously [7].

### Human leukemic cell lines and culture condition

Leukemic cell lines of B-cell lymphoid (RS411, 697, REH, Raji, IM9), T-cell lymphoid (HSB2, CEM119, Jurkat, Molt 3, SupT1), myeloid (KG1a, Kasumi-1, HL60, K562), natural killer (NK-92) lineages, and myeloma NCIH929 line were obtained from the American Type Culture Collection (ATCC; Manassas, VA, USA). These cell lines were cultured in Iscove modified Dulbeccco medium (IMDM; Invitrogen, Carlsbad, CA, USA) or RPMI 1640 medium (Invitrogen) containing 10% fetal bovine serum (FBS; Invitrogen) (20% for Kasumi-1 cells), 1 x Penicillin-Streptomycin (Invitrogen) accordingly to the manufacturer's instruction. Megakaryoblastic cell lines (MEG01, MO7e, CHRF288) were obtained and maintained as previous described [8]. The human embryonic stem cell (ESC) lines H9 (P48-53) and H14 (P44-68) were products of Wicell (Madison, WI, USA) and maintained as previously described [9].

### Over-expression of MKRN2 in CD34^+^ and K562 cells by lentivirus transduction

Full length makorin cDNA was subcloned into lentiviral vector (pLEF1αIG-MKRN2) (Fig A in File S1). The empty vector (pLEF1α-IG) was used as a control. The VSV-G pseudotyped lentivirus was produced by cotransfecting 293T cells with the transfer vector and three packaging vectors [10]. CD34^+^ or K562 cells were infected by the lentivirus at the multiplicity of infection (MOI) of 30. K562 cells were selected as the study model because K562 blasts are multipotential, hematopoietic malignant cells that could spontaneously differentiate into recognizable progenitors of the erythrocytic, granulocytic and monocytic lineages. In addition, we observed that *MKRN2* and *RAF1* were consistently and highly expressed in K562 and in primary myelocytic leukemia cells. Cells were first transduced for 16 hr, followed by 12 hr recovery in medium with 10% FBS and then transduced for a further 16 hr. Analysis and further manipulation was conducted 48 hr post transduction. The percentage of GFP expressing cells was monitored by flow cytometry using FACS Calibur flow cytometer and the CellQuest software (BD Biosciences), with 7-amino-actinomycin D (7-AAD) staining to gate out dead cells.

### Silencing of MKRN2 by nucleofection using shRNA

A set of 29 mer shRNA constructs targeting *MKRN2* (pGFP-V-RS-MKRN2, 4 unique sh cassettes in retroviral GFP vector) was introduced to inhibit the expression of *MKRN2* in primary CD34^+^ cells and K562 cells. pGFP-V-RS (retroviral GFP vector) and pGFP-V-RS-NE (non-effective 29-mer sh GFP cassette retroviral GFP vector) were used as control experiments. All Hush constructs were purchased from OriGene Technologies (Rockville, MD, USA). Briefly, 200 ng of each of the shRNA plasmids were used for nucleofection. Enriched CB CD34^+^ cells (2×10^4^/mL) and K562 cells (1×10^5^/mL) were transfected using the Human CD34^+^ Cell Nucleofection Kit and K562 Nucleofection Kit (Amaxa Biosystems, Koeln, Germany), respectively. After nucleofection, cells were allowed to grow for 48 hr prior to measurement of readout parameters. The stable suppression of *MKRN2* in K562 cells was maintained using Puromycin treatment (1 μg/mL; Invitrogen).

### Cell viability

Transduced cells from each treatment were plated in duplicate wells (12-well plates, Corning) with the appropriate culture conditions (starting at 2×10^4^ cells/mL) and splitted at a ratio of 1∶3 on day 6. Cells were counted daily by a hematocytometer under light-microscope, with trypan-blue staining (0.4%; Bio-Rad, Hercules, CA, USA) to exclude dead cells.

### Ex vivo expansion of transfected CD34^+^ cells

Enriched CD34^+^ cells at 2×10^4^/mL were expanded in IMDM containing 10% FBS (StemCell Technology, Vancouver, Canada), 0.1% BSA, thrombopoietin (TPO; 50 ng/mL), stem cell factor (SCF; 50 ng/mL) and Flt-3 ligand (FL; 80 ng/mL). All cytokines were products of Peprotech (Rocky Hill, NJ, USA). After 8 days, multilineage stem/progenitor cells in the expansion culture were quantified by further culture for 14 days in cytokine-enriched methylcellulose medium (StemCell Technology). The number of colony forming units (CFU) of the erythroid (BFU-E, CFU-E), myeloid (CFU-GM) and early mixed (CFU-GEMM) lineages was counted under a microscope.

### MTT assay

Effects of over-expression and inhibition of *MKRN2* on proliferation of K562 cells were assessed by the methabenzthiazuron (MTT) method. Cells (5×10^4^ per well) were seeded in duplicates onto a 24-well plate (Corning, NY, USA) and incubated with 100 μL MTT (5 mg/mL; Invitrogen) for 30 min at 37°C. The insoluble violet formazan crystals and cells were collected by centrifugation at 18,300×*g* for 10 min and dissolved in 100 μL dimethylsulphoxide (DMSO, Invitrogen). Absorbance was read at 570 nm. Duplicate measurements were determined in 3 independent experiments and expressed as percentage of the control.

### Reverse transcription and qPCR

Total RNA was extracted from cell cultures (1×10^6^/samples), peripheral blood MNC or bone marrow samples using Trizol reagent (Invitrogen). cDNA was synthesized from 1 μg of total RNA using the High Capacity cDNA Reverse Transcription Kit (Applied Biosystems, Foster City, CA, USA). qPCR analysis was carried out using human specific Taqman Gene Expression Assays (Applied Biosystems). These primer and probe sets (*MKRN2*, Hs00274055_m1 and *RAF1*, Hs00234119_m1) have been recommended for specific gene expression experiments because they detect the maximum number of transcripts for target genes. Results were expressed as relative to glyceraldehyde-3-phosphate dehydrogenase (*GAPDH*).

### Statistical analysis

The significance of growth or inhibitory effects exerted by over-expression or shRNA suppression of *MKRN2* in CD34^+^ cells and K562 cell line were determined by the paired-samples *t* test. The differences in *MKRN2* and *RAF-1* mRNA expression levels between normal and malignant hematopoietic cells, and leukemic cell lines were determined by independent samples *t* test. Correlations of *MKRN2* and *RAF*-1 in primary leukemic samples and normal bone marrow samples were analyzed by the Pearson Correlation test. All analyses were performed using SPSS for Windows 17 software (SPSS, Chicago, IL, USA). A *P* value of ≤0.05 was considered significant. Results are expressed as mean ± standard error of the mean (SEM).

## Results

### mRNA expression of MKRN2 in normal and malignant hematopoietic cells

**Figure 1:**
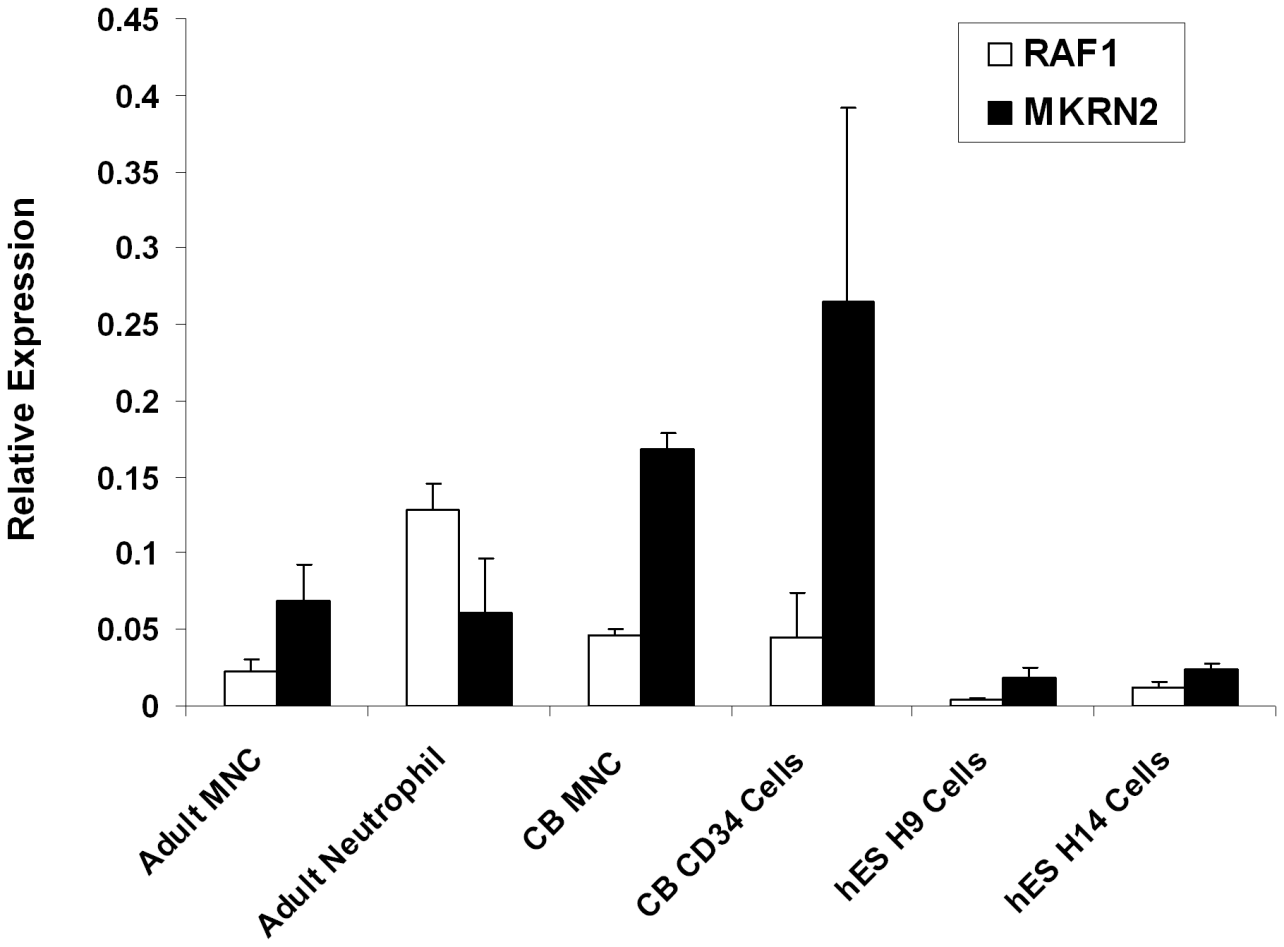
Expression of *MKRN2* and *RAF1* in primary hematopoietic cells and embryonic stem cell lines. Expression levels of *MKRN2* and *RAF1* mRNA were measured in adult and CB hematopoietic cells, enriched CD34^+^ stem/progenitor cells and human embryonic cell lines H9 and H14 by qPCR (n = 2–6). The Y-axis represents the expression level relative to *GAPDH*.

**Figure 2:**
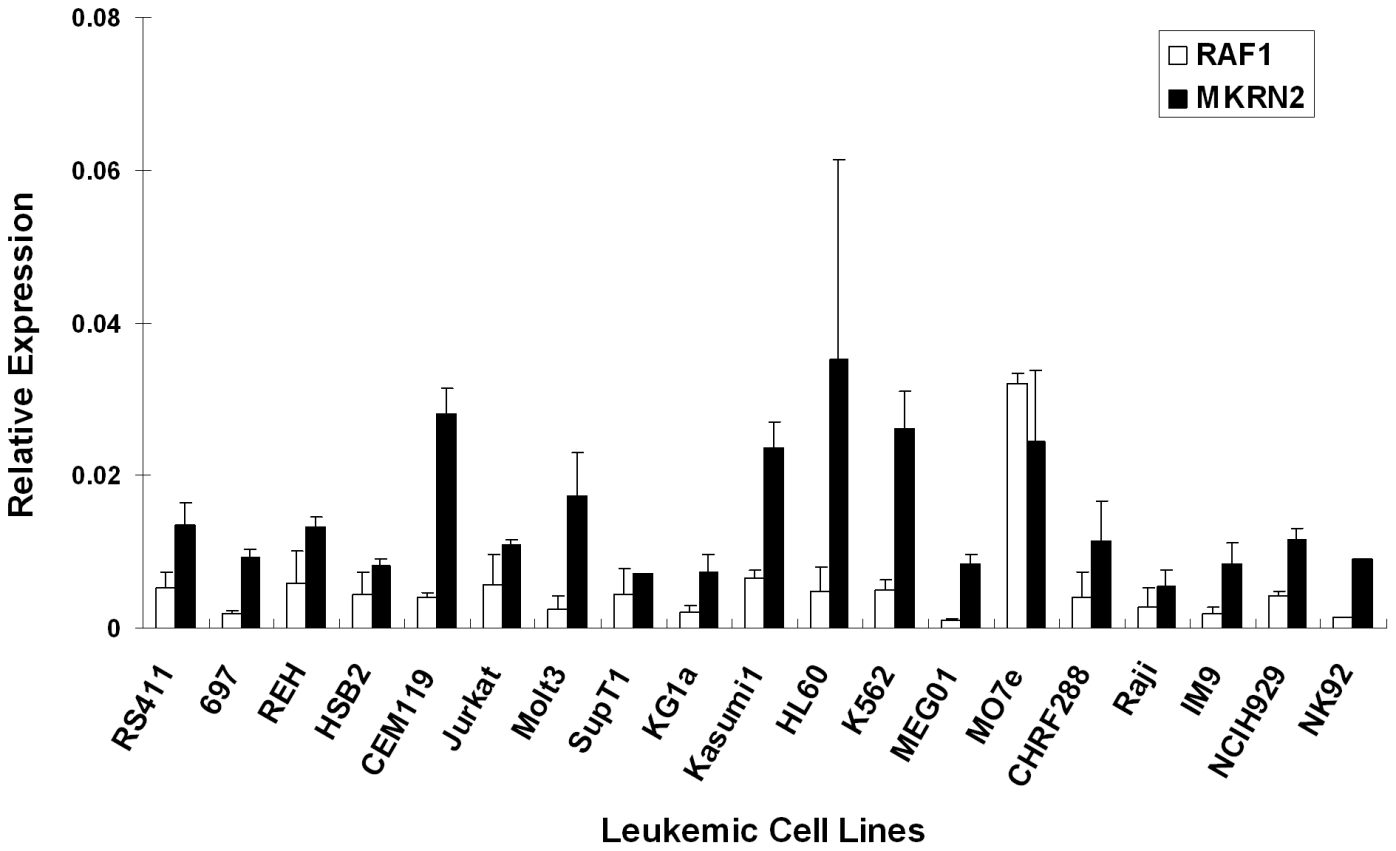
Expression of *MKRN2* and *RAF1* in leukemic cell lines. Expression levels of *MKRN2* and *RAF1* mRNA were measured by qPCR in leukemic cell lines of specific lineage subtypes. The Y-axis represents the expression level relative to *GAPDH*.

**Figure 3:**
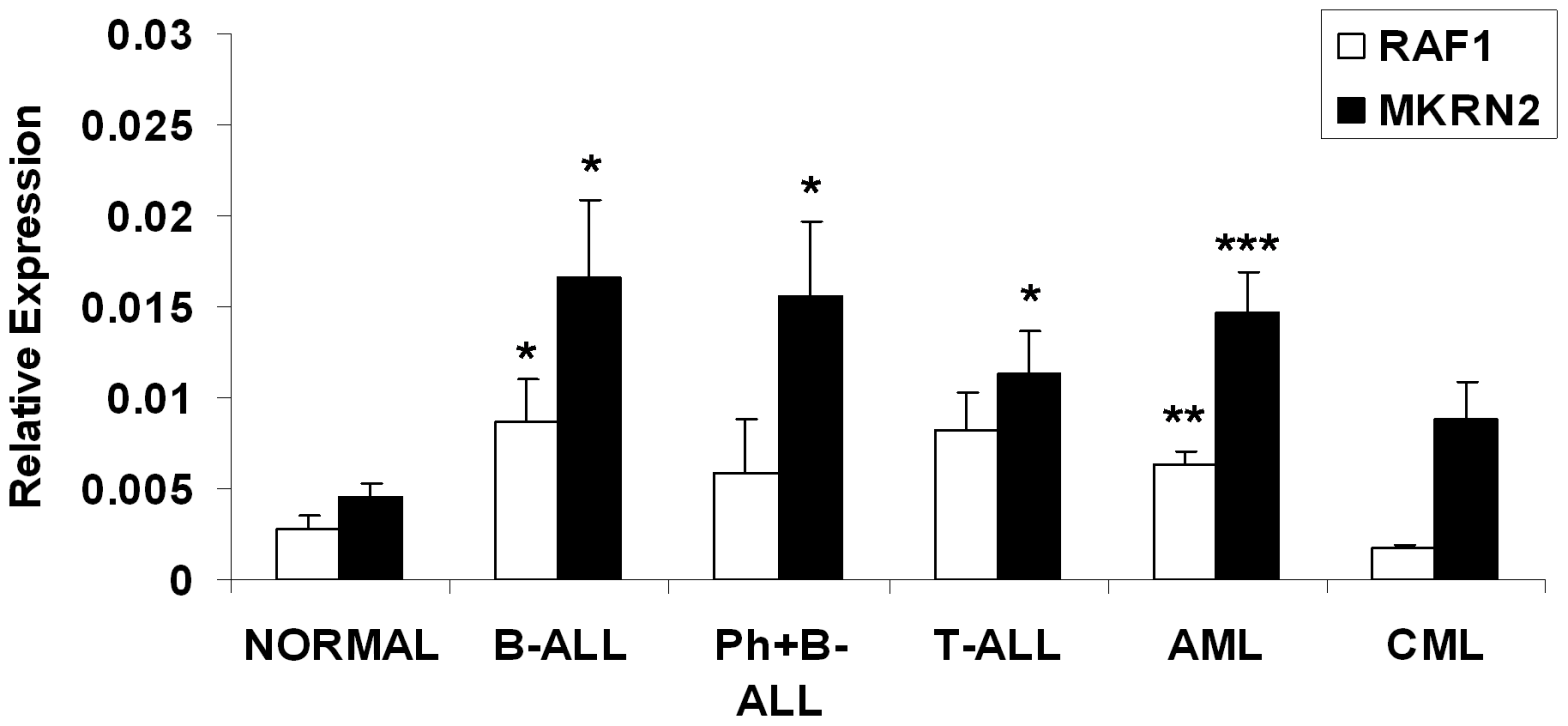
Expression of *MKRN2* and *RAF1* in primary human leukemic cells. Expression levels of *MKRN2* and *RAF1* mRNA were measured by qPCR in bone marrow cells collected from leukemic patients (Ph–B-ALL, n = 8; Ph+B-ALL, n = 7; T-ALL, n = 5; AML, n = 22 and CML, n = 11). The Y-axis represents the expression level relative to *GAPDH*. Expression levels of *MKRN2* and *RAF1* were compared with those of age-matched normal bone marrow cells (n = 9) (* *P*<0.05, ** *P*<0.01 and *** *P*<0.001). Ph  =  Philadelphia chromosome or *BCR/ABL* translocation.

By qPCR analysis, we observed ubiquitous expressions of *MKRN2* and *RAF1* in primary hematopoietic cells including adult MNC, neutrophils, cord blood MNC, enriched CD34^+^ cells, and human embryonic stem cell lines H9 and H14 (n = 2–6) (Fig 1). The expression levels are represented as relative to *GAPDH* (Y-axis, Fig 1–3). We also demonstrated expressions of *MKRN2* and *RAF1* in leukemia cell lines of B-ALL, T-ALL, AML, CML, NK and MK lineages (n = 2–3) (Fig 2). In primary leukemia samples obtained from BM of patients, we showed positive expressions of *MKRN2* and *RAF1* in B-ALL Philadelphia chromosome (*BCR/ABL* or Ph) positive and negative, T-ALL, AML and CML samples (n = 5–22) (Fig 3). The expression levels of *MKRN2* were generally higher in leukemia samples (*P*<0.05 in Ph–B-ALL, Ph+B-ALL, T-ALL and AML samples) compared with those in age-matched normal BM cells (n = 9), whilst *RAF1* was higher in Ph–B-ALL and AML samples (*P*<0.05). In all leukemia subtypes, there was no significant correlation between expression levels of *MKRN2* and *RAF1* (Fig B in File S1). In CML samples, there was no notable difference between *BCR/ABL* major (n = 8) and *BCR/ABL* minor samples (n = 3) in terms of *MKRN2* or *RAF1* mRNA expression (Fig C in File S1).

### Over-expression of MKRN2 in cord blood CD34^+^ cells

**Figure 4:**
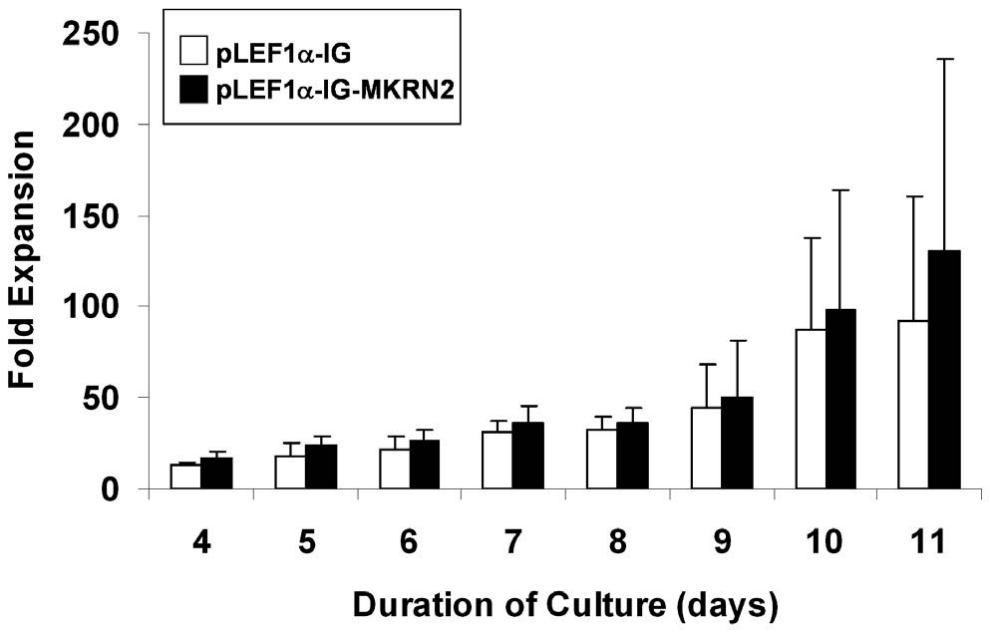
*Ex vivo* expansion of CD34^+^ cells overexpressing *MKRN2*. CD34^+^ cells were transduced with *MKRN2* cDNA subcloned into lentiviral vector (pLEF1α-IG-MKRN2) and cultured in expansion medium for 11 days (n = 3). The kinetics of expansion was not different between the *MKRN2*-transduced and control cells containing the empty vector (pLEF1α-IG).

**Figure 5:**
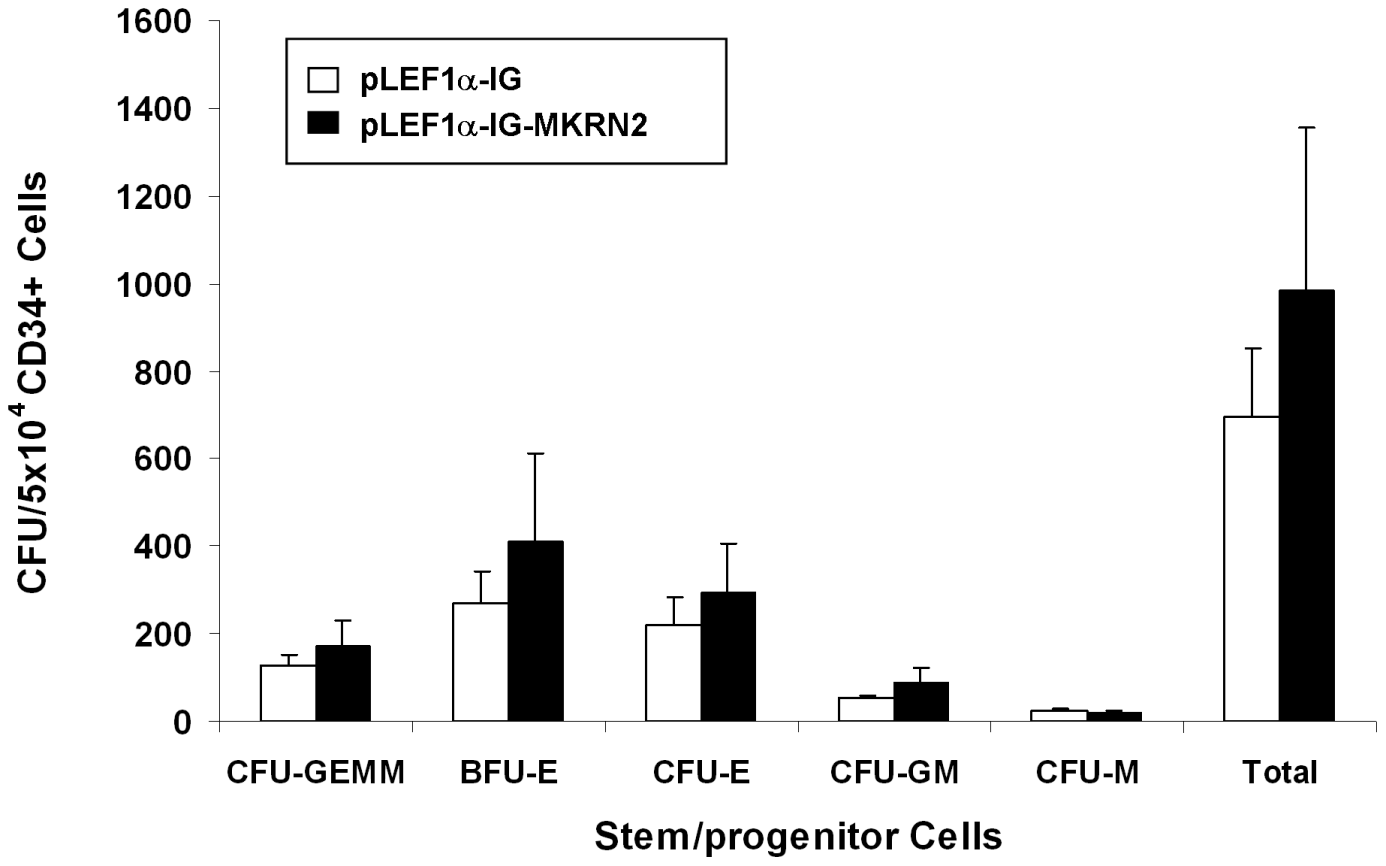
Colony forming capacity of *ex vivo* expanded CD34^+^ cells over-expressing *MKRN2*. CD34^+^ cells were transduced with *MKRN2* cDNA subcloned into lentiviral vector (pLEF1α-IG-MKRN2) and expanded for 8 days, and subjected to CFU culture for 14 days (n = 3). There was no difference between *MKRN2*-transduced cells and control cells containing the empty vector (pLEF1α-IG).

Lentiviral transduction of *MKRN2* resulted in 1.14±0.09 fold change of *MKRN2* mRNA and 0.93±0.27 fold change of *RAF1* in total CD34^+^ cells, relative to their respective levels in control cells containing the empty vector (n = 3). There were trends of increased cell expansion (Fig 4) and the number of multilineage stem/progenitor cells (Fig 5) after *ex vivo* culture of transfected cell in the presence of cytokine supplement. However, the differences were not statistically significant.

### ShRNA-silencing of MKRN2 in cord blood CD34^+^ cells

**Figure 6:**
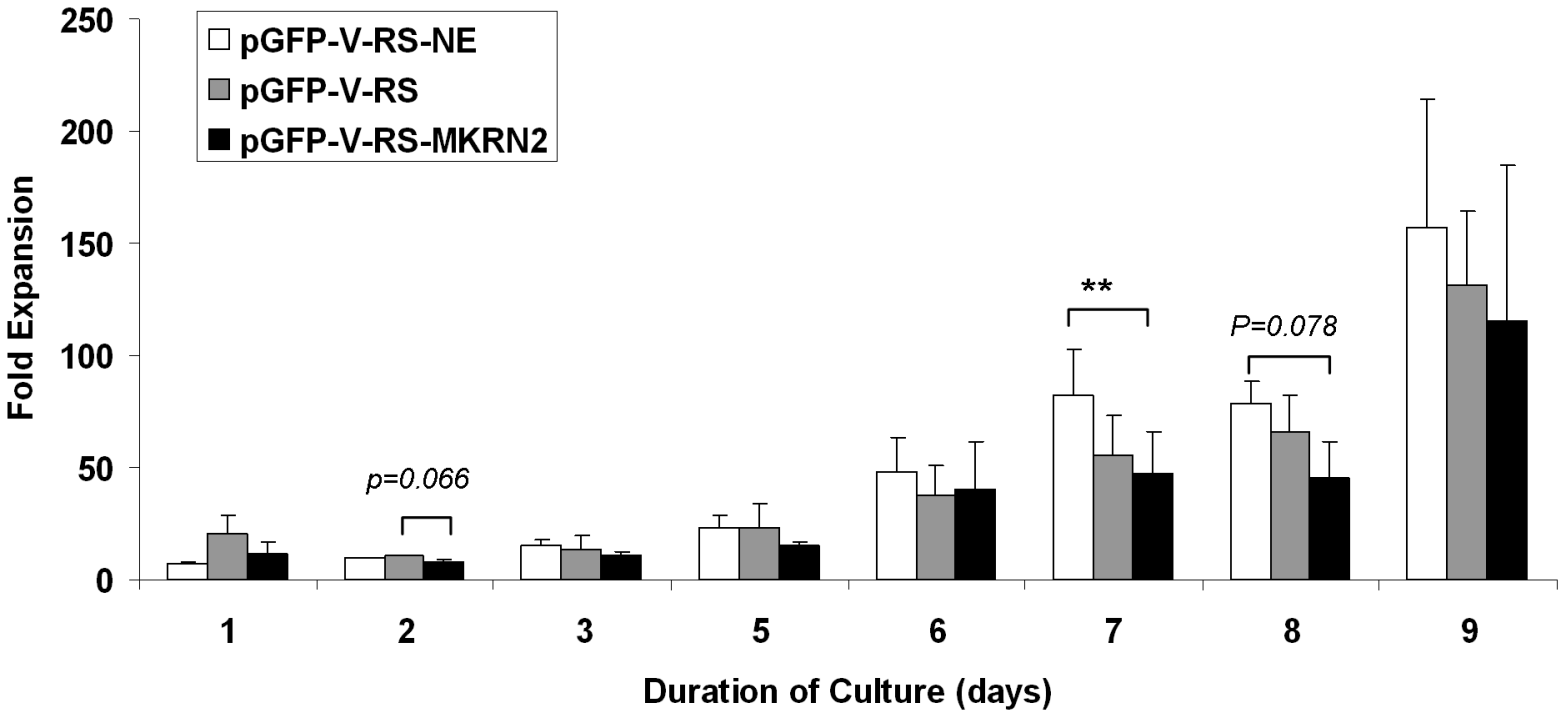
ShRNA-silencing of MKRN2 in cord blood CD34^+^ cells. *MKRN2* expression was down-regulated in CD34^+^ cells by nucleofection of shRNA. CD34^+^ cells expressing pGFP-V-RS-MKRN2 had lower expansion capacity in day 7 culture (*P* = 0.005; n = 4) and a trend of reduced expansion at days 2 and 8, compared with cells transfected with non-effective sh-pGFP-V-RS-NE or empty vector (pGFP-V-RS).

**Figure 7:**
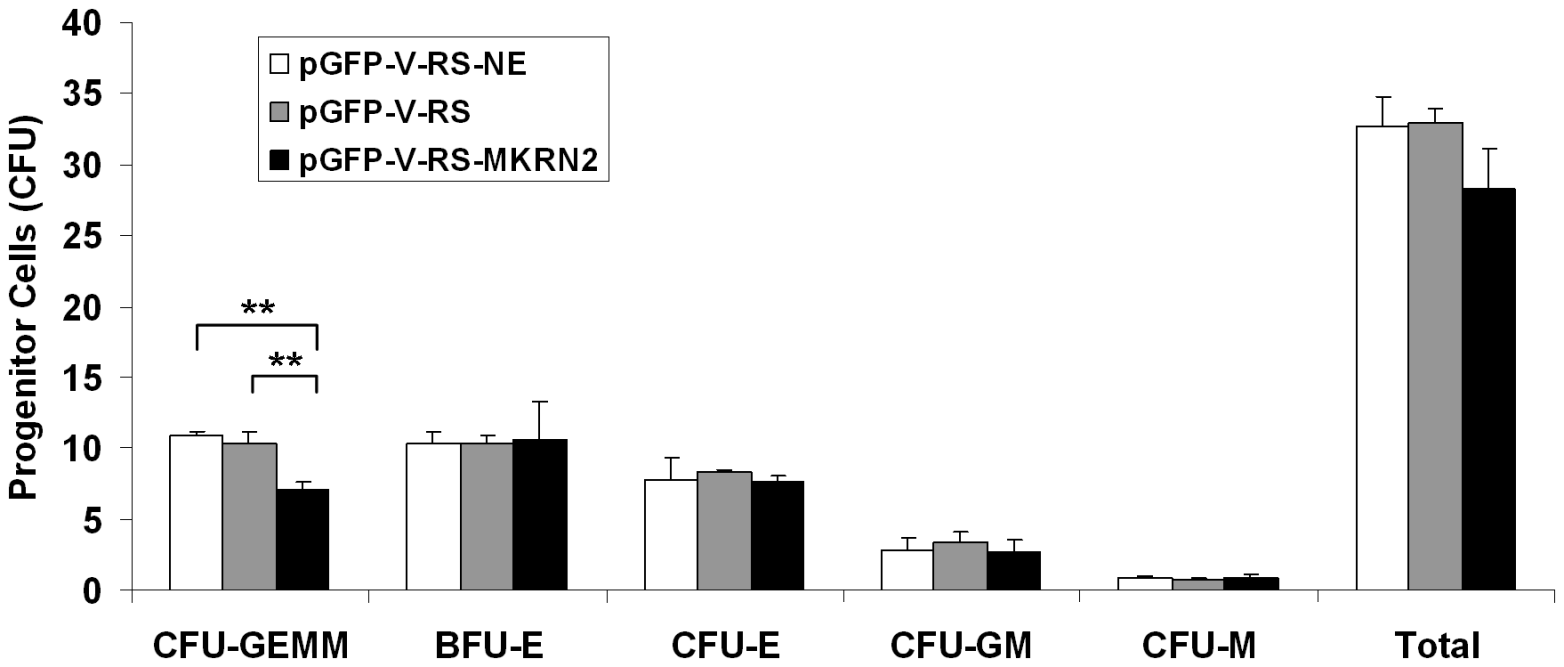
Colony forming capacity of *ex vivo* expanded CD34^+^ cells with silenced *MKRN2*. *MKRN2* expression was down-regulated in CD34^+^ cells by nucleofection of shRNA. CD34^+^ cells expressing pGFP-V-RS-MKRN2 had a lower level of CFU-GEMM, compared with cells transfected with non-effective sh-pGFP-V-RS-NE or empty vector (pGFP-V-RS) (** *P*<0.01; n = 3).

ShRNA inhibition of *MKRN2* resulted in reduction of *MKRN2* expression (0.66±0.12 fold vs. pGFP-V-RS; and 0.79±0.07 fold vs. pGFP-V-RS-NE) (Fig 6). *RAF1* expression in *MKRN2*-silenced cells was 1.28±0.31 fold compared with that in control pGFP-V-RS cells. CD34^+^ cells expressing pGFP-V-RS-MKRN2 had significantly lower proliferation capacity as shown in day 7 culture (*P* = 0.005) and a trend of reduced expansion at day 2 and day 8 cultures (n = 3), when compared with either non-effective sh cassette (pGFP-V-RS-NE) or empty vector (pGFP-V-RS) control cultures. The early stem/progenitor cells (CFU-GEMM) were also decreased in the pGFP-V-RS-MKRN2 nucleofected cell expansion culture (Fig 7).

### Over-expression of MKRN2 in K562 cells

**Figure 8:**
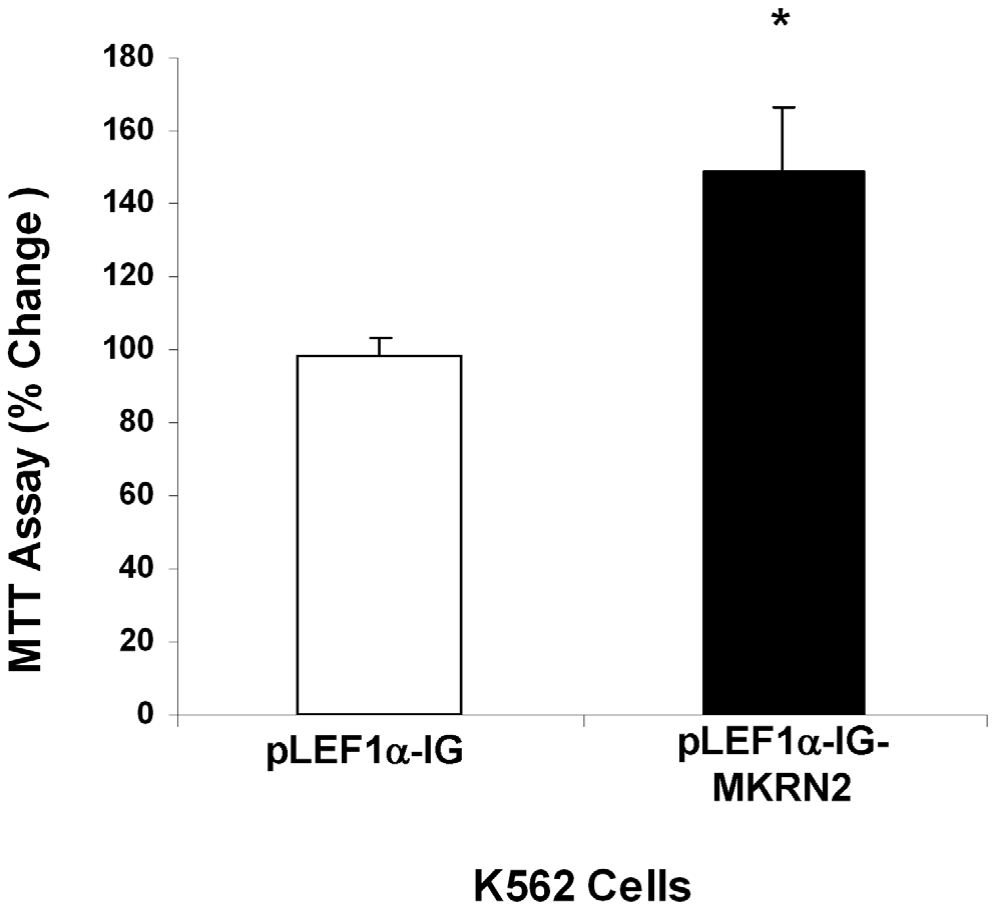
Proliferation capacity of K562 cells over-expressing *MKRN2*. K562 cells were transduced with *MKRN2* cDNA subcloned into lentiviral vector (pLEF1α-IG-MKRN2). MTT assay of pLEF1α-IG-MKRN2-transduced cells showed a significantly increased proliferation in culture, compared with pLEF1α-IG (empty vector) control cells (*P = 0.05; n = 3).

Lentiviral transduction of *MKRN2* in K562 cells resulted in 1.54±0.21 fold change of *MKRN2* mRNA expression compared with pLEF1α-IG control cells (n = 3). *RAF1* expression in *MKRN2*-tranduced cells was 1.06±0.03 fold of control cells. By flow cytometric analysis, 83±15.3% (range 52–98%) of pLEF1α-IG and 90±4.79% (80.1–95.2%) of pLEF1α-IG-MKRN2 transduced K562 cells expressed GFP (n = 3) (Fig D in File S1). MTT assay of pLEF1α-IG-MKRN2 transduced cells showed a significantly increased proliferation in culture, compared with that of the pLEF1-IG control cells (*P* = 0.05) (Fig 8).

### ShRNA-silencing of MKRN2 in K562 cells

At 2 days post-nucleofection, Sh-MKRN2-silenced K562 had 0.86±0.02 fold change of *MKRN2* expression and 1.39±0.27 fold change of RAF1 expression (n = 3). GFP positive cells ranged 62.2 – 85.3% of the total cell population. However, shRNA silencing of *MKRN2* did not reduce cell proliferation of K562 cells in culture (MTT assay).

## Discussion

Our data provided the first evidence on the ubiquitous expression of *MKRN2* in multi-lineage normal hematopoietic and leukemic cells, as well as its function on promoting CD34^+^ and K562 cell proliferation. In spite of the known conservation of the *MKRN2* gene through evolution, little has been reported on its role in any organism other than the anti-neurogenic activity in *Xenopus laevis* [3], [4]. Using shRNA silencing, we demonstrated the activity of *MKRN2* on promotion of CD34^+^ cell expansion to early progenitor cells, indicating its role on normal hematopoiesis. However, over-expression of *MKRN2* in CD34^+^ cells did not significantly affect cell expansion and lineage development, possibly because endogenous levels of MKRN2 protein were sufficient for its cell promoting functions. Upregulated expressions of *MKRN2* in primary leukemia cells prompted us to further investigate the effects of forced expression and silencing of *MKRN2* in the leukemic cell line K562. Again, we observed the stimulating activity of over-expressing *MKRN2* on K562 proliferation. In contrast to CD34^+^ cells, sh-silencing of *MKRN2* in K562 did not affect cell proliferation, indicating possible differences between the regulatory mechanism of *MKRN2* in CD34^+^ cells and leukemic cell line K562. Further evaluation of *MKRN2* gene manipulation on cell cycle regulation might reveal its specific mechanism on hematopoietic cell proliferation.

Due to the common sequence between *MKRN2* and *RAF1* in the antisense orientation, we suspected existence of a mutual regulatory mechanism between the two genes [11], [12]. RAF1, a protein closely associated with the RAP1, RAS, ERK and AKT pathways, plays multiple roles in hematopoietic cells [13]. It is required for growth factor-induced proliferation of normal hematopoietic and leukemic cells [14]. RAF1 is also implicated in drug resistance of *BCR/ABL* expressing leukemic cells [15]. In normal and leukemic cells, however, we only observed ubiquitous expressions of *MKRN2* and *RAF1*. They did not exhibit any convincingly significant correlation in their expression patterns. It is anticipated that a larger sample size of each leukemia subtype would be required to accurately address the relationship between *MKRN2* and *RAF1*, as well as between specific translocations such as *BCR-ABL*.

To our knowledge, there have been very few reports on the involvement of *MKRN2* in malignancy, except some microarray screening data on papillary thyroid cancer [16]. *MKRN1*, the most studied member of the *MKRN* family has been shown to participate in a variety of mechanisms such as RNA-II-dependent transcription [17], Oct-4 signaling in mouse embryonic stem cells [18], telomere length homeostasis in cancer cell lines [19], [20], polycystic kidney [21], ubiquitinase activity [22], and p14ARF-associated cellular senescence and gastric tumorigenesis [23]. Based on the ubiquitous expression and proliferative promoting activity of *MKRN2* in the various developmental windows of hematopoiesis, we suggest that *MKRN2* may play a house-keeping role on normal hematopoiesis. Our study has provided evidence that *MKRN2* might also be involved in the proliferation of human leukemic cells. Further knowledge on *MKRN2* interaction with known proto-oncogenes and involvement in leukemogenesis may lead to development of alternative treatment for the malignancy.

## Supporting Information

File S1 Figure A: *MKRN2* construct for lentiviral transduction. Figure B: Correlation of *MKRN2* and *RAF1* Expression in Leukemia Samples. Expression levels of *MKRN2* and *RAF1* mRNA, relative to *GAPDH*, in bone marrow cells collected from leukemic patients (Ph–B-ALL, n = 8; Ph+B-ALL, n = 7; T-ALL, n = 5; AML, n = 22 and CML, n = 11) and age-matched normal bone marrow donors (n = 9) were measured by qPCR and analyzed by Pearson correlation test. A positive correlation (*P* = 0.042) was observed in Ph+B-ALL samples. However, the correlation became insignificant when the one sample with extremely high expressions of both *MKRN2* and *RAF1* was excluded from analysis. Ph  =  Philadelphia chromosome or *BCR/ABL* translocation. Figure C: Expression of *MKRN2* and *RAF1* in CML patients with Major or Minor *BCR/ABL*. Expression levels of *MKRN2* and *RAF1* mRNA, relative to *GAPDH*, in bone marrow cells collected from CML *BCR/ABL* Major (n = 8) and Minor (n = 3) leukemic patients were measured by qPCR. There were no significant differences between the mRNA expression of either genes in the 2 subgroups of CML patients. Ph  =  Philadelphia chromosome or *BCR/ABL* translocation. Figure D: Flow cytometric analysis of K562 transduction with MKRN2-GFP. Representative flow cytrometric scatter plots of K562 cells lentiviral transduced with MKRN2-GFP. The empty vector GFP-IGV was used as a control. (A) Forward-scatter (x-axis) and side-scatter (y-axis) plot of K562 cells. R1 was gated for GFP expression analysis. (B) GFP expression (x-axis) and 7-AAD (y-axis, representing dead cells) of non-transduced cells. (C) K562 cells transduced with GFP-IGV control vector, showing 91.8% cells with GFP expression. (D) K562 cells transduced with MKRN2-GFP, showing 90.4% GFP-positive expression.
(DOC)
