# Supplementary material for: Ubiquitous Expression of MAKORIN-2 in Normal and Malignant Hematopoietic Cells and Its Growth Promoting Activity
Source: PLoS One. 2014 Mar 27;9(3):e92706. doi: 10.1371/journal.pone.0092706 (PMC3968021; doi:10.1371/journal.pone.0092706)
Supplement: File S1 — Figure A: MKRN2 construct for lentiviral transduction. Figure B: Correlation of MKRN2 and RAF1 Expression in Leukemia Samples. Expression levels of MKRN2 and RAF1 mRNA, relative to GAPDH, in bone marrow cells collected from leukemic patients (Ph–B-ALL, n = 8; Ph+B-ALL, n = 7; T-ALL, n = 5; AML, n = 22 and CML, n = 11) and age-matched normal bone marrow donors (n = 9) were measured by qPCR and analyzed by Pearson correlation test. A positive correlation (P = 0.042) was observed in Ph+B-ALL samples. However, the correlation became insignificant when the one sample with extremely high expressions of both MKRN2 and RAF1 was excluded from analysis. Ph = Philadelphia chromosome or BCR/ABL translocation. Figure C: Expression of MKRN2 and RAF1 in CML patients with Major or Minor BCR/ABL. Expression levels of MKRN2 and RAF1 mRNA, relative to GAPDH, in bone marrow cells collected from CML BCR/ABL Major (n = 8) and Minor (n = 3) leukemic patients were measured by qPCR. There were no significant differences between the mRNA expression of either genes in the 2 subgroups of CML patients. Ph = Philadelphia chromosome or BCR/ABL translocation. Figure D: Flow cytometric analysis of K562 transduction with MKRN2-GFP. Representative flow cytrometric scatter plots of K562 cells lentiviral transduced with MKRN2-GFP. The empty vector GFP-IGV was used as a control. (A) Forward-scatter (x-axis) and side-scatter (y-axis) plot of K562 cells. R1 was gated for GFP expression analysis. (B) GFP expression (x-axis) and 7-AAD (y-axis, representing dead cells) of non-transduced cells. (C) K562 cells transduced with GFP-IGV control vector, showing 91.8% cells with GFP expression. (D) K562 cells transduced with MKRN2-GFP, showing 90.4% GFP-positive expression. (DOC) [file pone.0092706.s001.doc]

**Supporting Information File S1**

**Figure A: *MKRN2* construct for lentiviral transduction**

**Figure B: Correlation of *MKRN2* and *RAF1* Expression in Leukemia Samples**

**Pearson Correlation (Ph+B-ALL) R^2^ = 0.60, *P* = 0.042**

**Figure C: Expression of *MKRN2* and *RAF1* in CML patients with Major or Minor *BCR/ABL***

**Figure D: Flow cytometric analysis of K562 transduction with MKRN2-GFP**
